# Variation of thermal conductivity of DPPC lipid bilayer membranes around the phase transition temperature

**DOI:** 10.1098/rsif.2017.0127

**Published:** 2017-05-24

**Authors:** Sina Youssefian, Nima Rahbar, Christopher R. Lambert, Steven Van Dessel

**Affiliations:** 1 Civil and Environmental Engineering Department, Worcester Polytechnic Institute, 100 Institute Road, Worcester, MA 01609, USA; 2 Chemistry and Biochemistry Department, Worcester Polytechnic Institute, 100 Institute Road, Worcester, MA 01609, USA

**Keywords:** lipid bilayers, DPPC, thermal properties, phase transition, molecular dynamics

## Abstract

Given their amphiphilic nature and chemical structure, phospholipids exhibit a strong thermotropic and lyotropic phase behaviour in an aqueous environment. Around the phase transition temperature, phospholipids transform from a gel-like state to a fluid crystalline structure. In this transition, many key characteristics of the lipid bilayers such as structure and thermal properties alter. In this study, we employed atomistic simulation techniques to study the structure and underlying mechanisms of heat transfer in dipalmitoylphosphatidylcholine (DPPC) lipid bilayers around the fluid–gel phase transformation. To investigate this phenomenon, we performed non-equilibrium molecular dynamics simulations for a range of different temperature gradients. The results show that the thermal properties of the DPPC bilayer are highly dependent on the temperature gradient. Higher temperature gradients cause an increase in the thermal conductivity of the DPPC lipid bilayer. We also found that the thermal conductivity of DPPC is lowest at the transition temperature whereby one lipid leaflet is in the gel phase and the other is in the liquid crystalline phase. This is essentially related to a growth in thermal resistance between the two leaflets of lipid at the transition temperature. These results provide significant new insights into developing new thermal insulation for engineering applications.

## 1. Introduction

Lipid membranes are a universal component of cellular organisms that separate the cell's interior from its exterior environment. They possess many unique features such as the ability to self-assemble in aqueous environments, incorporate various functional proteins or adapt to various environmental conditions. Phospholipids, the most abundant membrane lipids, consist of a polar hydrophilic head group, hydrophobic hydrocarbon tails (acyl chains) and a linkage that attaches head and tail groups [1]. The main phosphoglycerides are derived from phosphatidate by the formation of the ester or ether bonds between the phosphate group and the hydroxyl group of an alcohol [2]. The common alcohol moieties of phosphoglycerides are the amino acid serine, ethanolamine, choline, glycerol and inositol. The tails are typically fatty acids that differ in length and degrees of saturation [3]. In an aqueous medium, water molecules attract the head groups and repel the acyl chains. Hence, they form a sheet of lipid bilayer around the cell and create a barrier to ions and proteins from diffusing in or out of the cell. The interest in phospholipid thermodynamics and phase transitions has grown significantly due to their amphiphilic nature, chemical structure and strong thermotropic and lyotropic phase behaviour in an aqueous environment [4]. Previous studies show that the gel/fluid transition is different for each lipid, depending on the type of lipid, the length of the acyl chain, the degree of unsaturation along the chain and the type and nature of the polar head group [5,6]. An increase in acyl chain by two carbon atoms can raise the transition temperature by 10–20°C [7]. Adding one degree of unsaturation, can also increase the phase transition temperature by 10–20°C while adding two or more degrees of unsaturation does not lower the phase change temperature of the lipid [1,7]. In another study, it has been shown that at low temperature some bacteria replace phosphatidylethanolamine with phosphatidylglycerol or phosphatidylcholine head groups of the same acyl chain, in order to change the transition temperature of the lipid bilayer [8].

Molecular dynamics (MD) simulations of the phase behaviour of phospholipids using various modelling techniques revealed the underlying mechanism of this phenomenon. The investigation of the dynamic behaviour of phospholipids in the gel phase suggested that not only the lipid–lipid interactions but also lipid–water interactions play a critical role in the phase transition from the fluid phase to the gel phase [9]. The main mode of transport in the gel phase was found to be hopping which is seen in both the translational and rotational dynamics. MD simulations of dipalmitoylphosphatidylcholine (DPPC) and 1,2-dipalmitoyl-sn-glycero-3-phosphoethanolamine (DPPE) lipid bilayer showed that in spite of their similar chemical structures, the transformation process from a gel to a liquid-crystalline state is different [10]. This is due to the smaller head group of DPPE. Coarse-grained MD simulations of the transformation between a gel and a fluid phase in DPPC bilayers showed that the critical step in the transformation process is the nucleation of a gel cluster consisting of 20–80 lipids in both leaflets simultaneously [11]. These domains rapidly grow by converting the fluid phase into a gel phase. Lipid lateral diffusion rates are of the order of 10^−9^ cm^2^ s^−1^ which is two orders of magnitude less than that of the fluid phase.

The variations of lipid bilayer structure lead to changes in lipid membrane thermal properties. Although there have been different studies on the thermal conductivity of DPPC lipid bilayers [4,12,13], the mechanism by which the thermal conductivity of DPPC lipid bilayer varies due to changes in the structure around phase transition temperature is unknown. In this study, we report on the thermal properties of hydrated DPPC bilayers using MD simulations. Here we calculate temperature-dependent thermal conductivity values for DPPC above, at, and below the critical phase transition temperatures, in order to better understand system behaviour at these transitions. The objective of this effort is to obtain detailed information about membrane thermal conductivity phenomena, which can be used for heat transfer in whole cells or cell assemblies. The characterization of heat transfer phenomena in lipid membranes also renders new insights that can be leveraged towards developing new thermal insulation materials and heat transfer systems for various engineering applications, for example, for use in buildings, electronic devices or medical applications.

## 2. Material and methods

**Figure 1.**
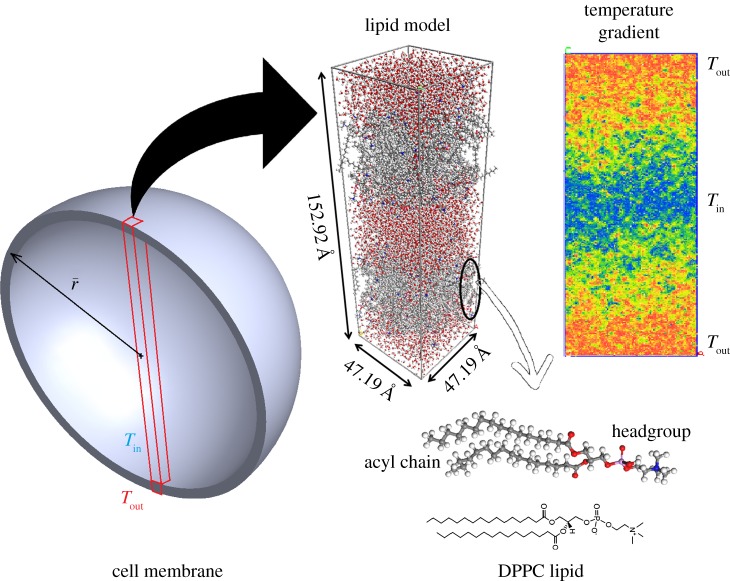
Heat transfer across the cell membranes: a model that consists of two lipid bilayers with 72 DPPC lipids was created to investigate the energy flow across the lipid bilayer in a cell membrane. The imposed heat flux creates a temperature gradient from a low temperature inside the cell (*T*_in_) to a high temperature outside the cell (*T*_out_).

An atomistic model of DPPC bilayer with 72 lipid and 2560 water molecules was used for the MD simulations with the polymer consistent forcefield, which was developed based on CFF91 for application to organic materials including lipids [14–16]. In order to relax the artificial energies of the system, the DPPC model was subjected to a series of dynamics simulations. Initially, the canonical ensemble (NVT) dynamics was carried out for 0.3 ns at 300 K, followed by 0.3 ns of isothermal–isobaric ensemble (NPT) dynamics at a temperature of 400 K and a pressure of 1 atm. In this stage, the atoms interact with each other at a higher kinetic energy. This helps the convergence of the system. Finally, the system cooled down to the desired temperature in NPT dynamics for 0.3 ns at 320 K and 1 atm (more details can be found in [17]). The outcome of this process is a cell with a cross-section of 47.245 × 42.319 Å^2^ that results in an area per lipid of 62.5 Å^2^. This is in good agreement with the experimental value of 62 to 64 Å^2^ at 323 K reported by previous studies [11,18]. To reduce the calculation error caused by selecting a specific configuration, two different configurations of DPPC model were obtained using the annealing process. Annealing process is a metaheuristic algorithm for locating a good approximation to the global minimum of a given function in a large search space. In this process, the lipid bilayer was heated up to 400 K and cooled down slowly to 300 K at a rate of 1 K ps^−1^ (for 4 × 10^5^ steps) for two cycles. During this process, at high temperatures, large energy increases are acceptable, allowing the system to explore a vast region of the search space. As the temperature is very low, the system is forced to stay in the local minimum in the current region of the search space. The advantage of this method is to enable the system to escape from local minima and search for better configurations. In each cycle, the configuration with minimum energy was selected for further calculation of the thermal energy transports across the lipid layer immersed in water molecules (figure 1).

### 2.1. Phase transition temperature

The lipid model was used to calculate the phase transition temperature of DPPC. In this process, a metaheuristic algorithm was used for locating a good approximation of the bilayer structure at each temperature. The system was heated up to 400 K and cooled down slowly to 200 K at 0.2 K ps^−1^, while the temperature and pressure were controlled by the Nose thermostat and Berendsen barostat. During this process, 23 structures at random temperatures were chosen to plot the specific volume versus temperature curve. The phase transition temperature was obtained from the temperature at which the slope of the curve changes.

### 2.2. Thermal conductivity

The lipid model was used to calculate the conductivity of the lipid bilayer across the membrane using a non-equilibrium MD method, in which an energy flux is imposed on the system. There are two variants of the imposed flux method: the reverse non-equilibrium molecular dynamics (RNEMD) method [19] and the imposed flux method [20]. In the RNEMD method, the energy exchange occurs by replacing the kinetic energy of the hottest particle in the cold layer and the coldest particle in the hot layer. The energy Δ*E* is, therefore, variable and requires averaging over many exchanges. In the imposed flux method the energy, Δ*E*, is fixed, and involves all particles in the hot and cold layers. This constant energy is subtracted from the energy of the particles inside the cold layer, and then added to the particles in the hot layer. Hence, a constant heat flux per unit area (*J*) is imposed between two layers that can be calculated by2.1
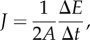
where *A* is the area perpendicular to the flux direction, and the factor 2 is due to the periodic boundary conditions, since an amount of *ΔE*/2 flows in or out of either sides of the layer. The energy modification of each layer is done by rescaling the velocities of the particles inside the layers [13,20,21]. Although the velocity scaling is a disturbance to the kinetic energy of the system, it does not significantly modify the local thermal equilibrium of the hot and cold layers [22,23]. Hence, this method conserves the total linear momentum of the hot and cold layers in addition to the total energy of the system. Finally, the thermal conductivity (*K*) is calculated from the ratio of the heat flux to the resulting temperature gradient (d*T*/d*z)*,2.2
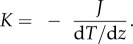


The lipid model was divided into 100 layers between which the constant energy exchange occurs. The system was allowed to exchange heat between layers for 2 ns to reach equilibrium.

From the calculated heat flux in the system and ensuing temperature gradients of layers (Δ*T*), the thermal resistances (*R*) were calculated by2.3
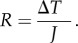


The thermal resistance at the interface of *i* and *j* layers (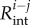) was calculated by2.4
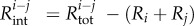
where 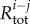 is the total thermal resistance between *i* and *j* layers and *R_i_* and *R_j_* are the thermal resistance of each layer.

### 2.3. Molecular interactions

To investigate the effects of molecular interactions at water/lipid and lipid/lipid interfaces on the thermal properties of the bilayer membranes, NVT dynamics at 300 and 350 K were performed on DPPC models for 1 ns. The resulting trajectories were used to calculate the area/lipid and adhesion energies at each temperature. The adhesion energy (*E*_adh_) is computed from the total energy (*E*_tot_) minus the sum of the energy of the two layers (*E*_1_ + *E*_2_).

## 3. Results and discussion

**Figure 2.**
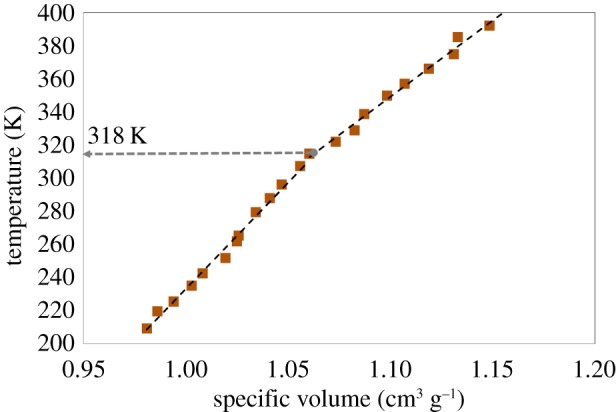
The phase transition temperature is computed from the variation in the specific volume of the DPPC bilayers as a function of temperature. The change in slope of the diagram at 318 K indicates a transition from liquid-crystalline phase to gel phase at the phase transition temperature.

Figure 2 shows the variation of specific volume of the DPPC bilayers with temperature. As the temperature rises, the specific volume increases. The rate of the increase, however, depends on the structure of the system. If the bilayer is in the gel phase, the rate of the variation is higher than the bilayer in the crystal-liquid phase. Therefore, the temperature at which the rate of specific volume changes defines the phase transition temperature. The simulation results of the DPPC model estimate the transition temperature of DPPC at about 318 K, which is in excellent agreement with the experimental results at 315 K [24,25].

**Figure 3.**
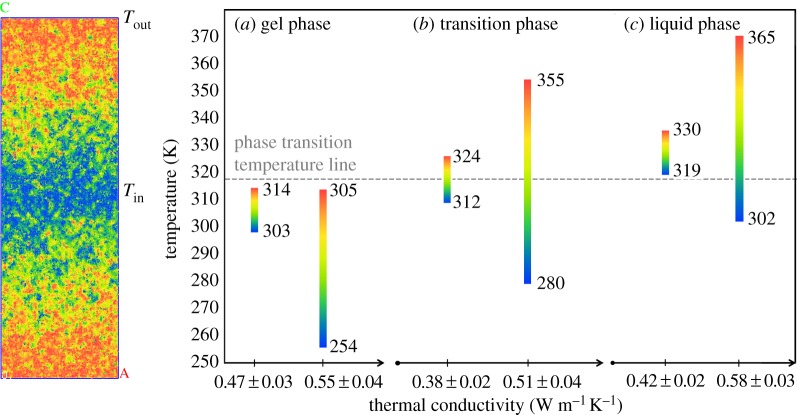
Thermal conductivity of the DPPC bilayer as a function of temperature for different temperature gradients when (*a*) both leaflets are in the gel phase, (*b*) one leaflet is in the gel phase and the other one is in the liquid crystalline phase and (*c*) both leaflets are in the liquid crystalline phase. The thermal conductivity of the DPPC bilayer is clearly higher at larger temperature gradients. The thermal conductivity of DPPC is lowest when leaflets are in different phases.

Through the phase change transition, many properties of the DPPC bilayer alter including the thermal properties. To elucidate the variation of the conductivity of the DPPC bilayer with temperature, three distinct stages were thoroughly studied. In the first stage, the temperatures of both lipid leaflets are lower than the phase transition temperature (gel phase). In the second stage, the temperature of one leaflet is above the transition temperature (gel phase) and the temperature of the other is below the phase transition temperature (liquid crystalline). Finally, in the third stage, the temperature of both leaflets is above the transition temperature. In each stage, two different temperature gradients were applied to the system, one with a temperature difference of approximately 16 K and the other with a temperature difference of approximately 70 K. Figure 3 presents the variation of thermal conductivity at different phases and temperature gradients. Regardless of the DPPC phase, the higher temperature gradient causes larger thermal conductivity in the lipid bilayer. In the gel phase, at low-temperature gradients the thermal conductivity is about 0.47 ± 0.03 W m^−1^ K^−1^ whereas at high-temperature gradients the value is calculated as 0.55 ± 0.04 W m^−1^ K^−1^. At the transition and liquid crystalline phases, the thermal conductivity varies from 0.38 ± 0.02 to 0.51 ± 0.04 W m^−1^ K^−1^, and from 0.42 ± 0.02 to 0.58 ± 0.03 W m^−1^ K^−1^, respectively. Although, to our best of knowledge, this is the first study on the temperature-gradient dependence of the thermal conductivity and we do not have experimental data to compare with, the calculated values are in the range of different lipid bilayer thermal conductivity measurements. The data obtained from the heat conductance of mammalian blubber which is a lipid-rich collagen fibre-laced material [26], shows that the heat conductivities are in the range of 0.21–0.31 W m^−1^ K^−1^ [27]. Also, MD simulations on the DPPC bilayer shows that for a temperature gradient of 7 K the thermal conductivity of the lipid bilayer is around 0.25 W m^−1^ K^−1^ [12].

Comparing thermal conductivities of different phases suggests that at the transition phase, thermal conductivity is the lowest. When DPPC goes from the gel phase to the transition phase with a low-temperature gradient, the thermal conductivity of DPPC decreases from 0.47 to 0.38 W m^−1^ K^−1^. Finally, thermal conductivity rises to 0.42 W m^−1^ K^−1^ when DPPC is in the liquid crystalline phase. The same trend is observed for the higher temperature gradients.

**Figure 4.**
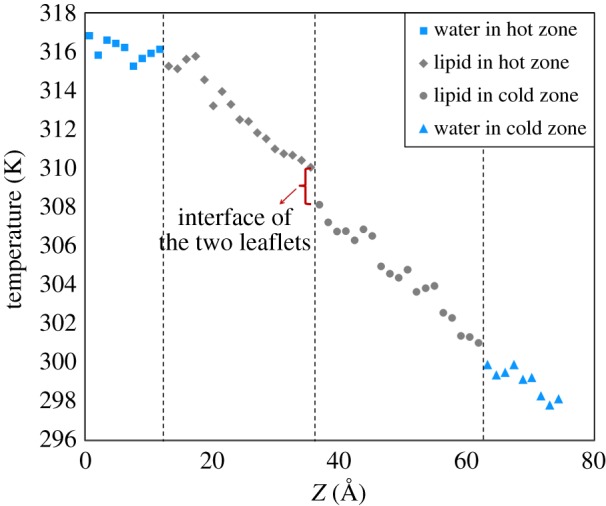
The variation of the temperature across the lipid bilayer from high temperature outside the cell (at 0 Å) to low temperature inside the cell (at 74.37 Å). The diagram has three points at which the temperature drops unexpectedly due to interfacial thermal resistance between the water molecules and head groups, and between the acyl chains of two different leaflets. The thermal resistance between acyl groups at 36 Å appears to be larger than the thermal resistance between the water molecules and head groups.

The temperature-gradient profiles across the lipid bilayer were also studied to understand the participation of each layer into the thermal conductivity of DPPC bilayers. Figure 4 illustrates a typical temperature-gradient profile of the lipid system. This profile shows four different regions that are distinguishable by the change in the slope of the diagram. The first and last regions represent the water molecules in the hot and cold temperature regions, respectively. From the slope of the diagram and energy flux of this system (904 MW m^−^^2^), the thermal conductivity of water at 316 and 297 K were calculated to be about 0.60 and 0.51 W m^−1^ K^−1^, respectively. These are in good agreement with experimental data that estimate the thermal conductivity of water to be around 0.63 and 0.60 W m^−1^ K^−1^ at 313 and 293 K, respectively [28]. The two middle layers represent the top and bottom layers of DPPC bilayers. These two have almost the same thermal conductivity which is estimated to be 0.39 W m^−1^ K^−1^. This indicates that lipid layers with lower thermal conductivity play major roles in controlling the thermal conductivity. One piece of information that is missing from these results is the amount of energy that is dissipated at the interface of water/lipid or lipid/lipid layers. In figure 4, there is a discontinuity at the interface of two layers due to the thermal resistance of the interface. The thermal resistance of the interface is thought to originate from the non-bonded interactions between atoms at the interfaces of two layers. Temperature-gradient profiles were used to estimate the thickness, and eqn (2.4) was used to compute the thermal resistance of the interface between different layers of the DPPC lipid bilayers.

**Figure 5.**
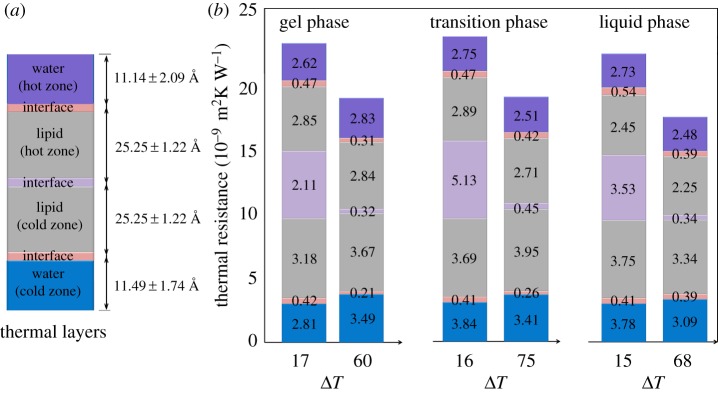
(*a*) Schematic of the thermal layers in the DPPC lipid bilayer system. The water and lipid layers are approximately 11.31 and 25.25 Å, respectively. (*b*) Schematic representation of the thermal resistance of each layer in the system. At lower temperature gradients, the overall thermal resistance of the DPPC bilayers is higher due to larger interfacial resistance between the acyl chains. The interfacial thermal resistance increases when the two leaflets are in different phases.

Figure 5*a* shows a thermal model for DPPC lipid bilayers with the estimated thicknesses of the different components. These results were calculated by averaging the thicknesses obtained from temperature-gradient profiles. Figure 5*b* presents the calculated thermal resistance of the layers and the interfaces, in different phases, for different temperature gradients. These results suggest that regardless of the DPPC phase, applying a higher temperature gradient, results in a lower thermal resistance.

Comparing the interfacial thermal resistances of lipid bilayers with that of chemical groups of organic molecules such as water–protein reveals more information about the thermal properties of cell membranes. The interfacial thermal resistances between water and proteins such as myoglobin, green fluorescence protein and Ca^2+^–ATPase protein are around 3 × 10^−9^ m^2^K W^−1^ [29–31] whereas this value for water–lipid is around 0.45 × 10^−9^ m^2^K W^−1^. Hence, the interfacial thermal resistance between water–lipid is an order of magnitude less than the thermal resistance at the interface of water–protein. On the other hand, while the interfacial resistance between lipid–lipid at high-temperature gradients is still an order of magnitude less than water–protein thermal resistance, at low-temperature gradients this value (approx. 5 × 10^−9^ m^2^K W^−1^) is comparable to the values found for interfacial resistance between water–protein. This indicates that the energy flow at the interface of the water–lipid is facilitated by the strong interaction energies between water molecules and polar head groups of DPPC.

Comparing the thermal resistance of the layers and interfaces at high- and low-temperature gradients indicated that a drop in the thermal resistance between acyl chains is the major factor in the decrease of thermal resistance. When DPPC is in the gel phase and the applied temperature difference is 17 K, the interfacial thermal resistance between the two layers was calculated to be about 0.49 × 10^−9^ m^2^K W^−1^ whereas for a 60 K temperature difference, the same thermal resistance was calculated to be 0.32 × 10^−9^ m^2^K W^−1^. This holds true for the thermal resistance between two lipid layers in the transition and liquid crystalline phases. In contrast, thermal resistance between water and DPPC head groups does not change significantly with variation of temperature gradients. The thermal resistance at the interface of water molecules and DPPC head groups is around 0.40 × 10^−9^ m^2^K W^−1^ in different phases and different temperature gradients.

When the system is in the transition phase, the overall thermal resistance of the DPPC lipid bilayer is at its highest. At low-temperature gradients, when the system goes from gel to gel–liquid phase the total resistance of the system slightly increases from 15.5 to 19.1 × 10^−9^ m^2^K W^−1^. Once it reaches the liquid crystalline phase, thermal resistance decreases to 17.3 × 10^−9^ m^2^K W^−1^. The same trend is observed for high-temperature gradients. This small increase is originated from a growth in the interface thermal resistance between acyl chains of the two lipid leaflets. The resistance between two layers of lipid increase from 2.11 × 10^−9^ to 5.13 × 10^−9^ m^2^K W^−1^, and it decreases to 3.53 × 10^−9^ m^2^K W^−1^ when DPPC goes from a gel to a liquid crystalline phase.

**Figure 6.**
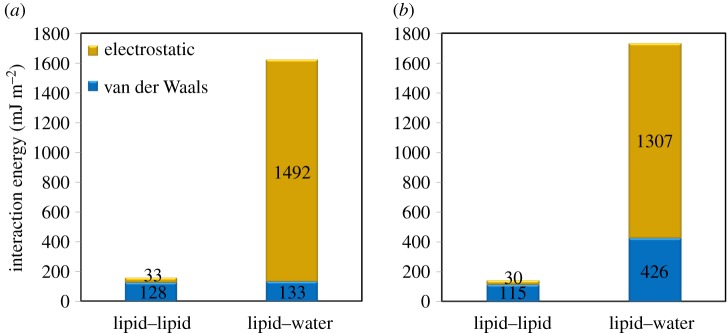
The interaction energies between acyl groups of two lipid leaflets, and the interaction energies between water molecules and head groups at (*a*) 300 K and (*b*) 350 K. The interaction energies between acyl groups are low due to the weak electrostatic energies between the acyl groups. The interaction energies between water molecules and head groups, on the other hand, are high because of the high polarity of phosphatidylcholines and water molecules. Low interaction energies between acyl chains increase the thermal resistance between the two DPPC layers.

Figure 6 presents the interaction energies between water molecules and head groups with interaction energies between acyl chains of the two leaflets at 300 and 350 K (figure 6*a* and *b*, respectively). The overall interaction energies between water molecules and lipid head groups at both temperatures are around an order of magnitude larger than that of the interaction energies between the lipid leaflets. The strong interactions between water molecules and head groups come from the strong electrostatic energies, due to high polarity of phosphatidylcholines and water molecules. The observed trends of the interfacial resistance on Figure 5 are attributed to a combination of strong cross-interface intermolecular interactions and good thermal coupling via soft vibration modes present at the interfaces [32]. Therefore, the strong interactions between water molecules and head groups can be one of the factors that facilitate the energy transfer between these particles. In contrast, acyl chains are non-polar fatty acids that mainly interact with each other by weak van der Waals forces. Hence, similarly, the interfacial resistance between two acyl chains can be affected by the poor energy transfer at the interface of the two layers. The influence of temperature on the interaction energies appears to be negligible.

**Figure 7.**
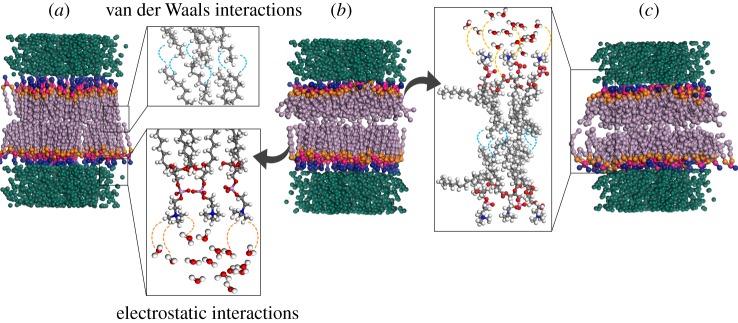
Three temperature-dependent nanostructures of DPPC lipid bilayers (*a*) both leaflets are in the gel phase, (*b*) the top leaflet is in the liquid crystalline phase while bottom leaflet is in the gel phase and (*c*) both leaflets are in the liquid crystalline phase. Due to weak van der Waals interactions between acyl chains of the two leaflets, the thermal resistance at the interface of two layers of DPPC bilayers is larger than the thermal resistance between the water and head groups. The thermal resistance significantly decreases when the temperature gradient increases. In (*b*) the thermal resistance between two leaflets is slightly higher than (*a*) and (*c*).

The thermal property of the DPPC bilayer is highly influenced by the thermal resistance between acyl chains of the two leaflets (figure 7). Since the interaction energies between acyl chains of the two leaflets are weak van der Waals interactions, they are subjected to more variations than strong electrostatic energies between water molecules and phosphatidylcholines of the head groups. The interaction energy at the interface of the two leaflets varies with the change in the nanostructure and arrangement of DPPC bilayers at the phase transition temperature and alters the thermal conductivity of the DPPC lipid. Figure 7 shows the three possible conformations of DPPC nanostructure at the phase transition temperature. In conformation I, both leaflets are in the gel phase (figure 7*a*). In conformation II, both leaflets are in the liquid crystalline phase (figure 7*c*) and in conformation III, one leaflet is in gel phase and the other leaflet is in liquid crystalline phase (figure 7*b*). When the leaflets are in different phases, due to weak interaction energies between the two leaflets and the growth in thermal resistance at the interface of acyl chains the overall thermal conductivity decreases.

The results also show that the thermal properties of the DPPC bilayer is gradient dependent. At higher temperature gradients, the thermal resistance between the two leaflets of the DPPC bilayer is significantly smaller than the thermal resistance at lower temperature gradients. The interaction energies decrease when the temperature gradient is low. This leads to a significant drop in phonon transport and an overall decrease in the thermal conductivity of the DPPC bilayer.

## 4. Conclusion and future work

In this paper, non-equilibrium MD simulations were used to calculate thermal conductivities of DPPC bilayers at different phases. To this end, the DPPC model was subjected to low and high-temperature gradients at gel, gel–liquid transition and liquid crystalline phases. The results show a remarkable property that, regardless of the DPPC phase, higher temperature gradients cause larger thermal conductivity in the lipid bilayer. Comparing thermal conductivities in different phases suggests that at the transition phase, thermal conductivity has the lowest value. The analysis of thermal resistance at the interfaces between layers of the system suggested that the thermal resistance between acyl chains of two DPPC leaflets is the main mechanisms of thermal conductivity variation. The growth of the thermal conductivity at higher temperature gradients is due to the decrease in the thermal resistance between acyl chains. At the transition phase, the thermal resistance between the acyl chains is at its highest, which results in an overall decrease in thermal conductivity. Since the major interaction energy between water molecules are the relatively strong electrostatics energy, the thermal resistance between these two layers is quite low. The dominant interaction energy between acyl groups are due to weak van der Waals forces, and thus the energy transport between acyl groups is weak. At the transition phase, as one leaflet is in the gel phase and the other is in the liquid crystalline, the interactions between acyl groups are minimum. This leads to a high thermal resistance between the two DPPC leaflets. These results render significant new insights into developing new thermal insulation for engineering applications. The insight from this research can also be expanded to address other biological applications such as understanding the lipid raft formation in which lipid bilayers are combined with protein receptors, organized in glycolipoprotein microdomains [33,34].

## Data accessibility

Research data of this article can be accessed upon request from the corresponding author.
